# Registered nurse, healthcare support worker, medical staffing levels and mortality in English hospital trusts: a cross-sectional study

**DOI:** 10.1136/bmjopen-2015-008751

**Published:** 2016-01-04

**Authors:** Peter Griffiths, Jane Ball, Trevor Murrells, Simon Jones, Anne Marie Rafferty

**Affiliations:** 1 National Institute for Health Research Collaboration for Leadership in Applied Health Research and Care (CLAHRC) Wessex, Southampton, UK; 2 University of Southampton, Southampton, UK; 3 Karolinska Institutet, Stockholm, Sweden; 4 Florence Nightingale Faculty of Nursing and Midwifery, King's College London, London, UK; 5 Department of Population Health, New York University School of Medicine, New York, New York, USA

## Abstract

**Objectives:**

To examine associations between mortality and registered nurse (RN) staffing in English hospital trusts taking account of medical and healthcare support worker (HCSW) staffing.

**Setting:**

Secondary care provided in acute hospital National Health Service (NHS) trusts in England.

**Participants:**

Two data sets are examined: Administrative data from 137 NHS acute hospital trusts (staffing measured as beds per staff member). A cross-sectional survey of 2917 registered nurses in a subsample of 31 trusts (measured patients per ward nurse).

**Outcome measure:**

Risk-adjusted mortality rates for adult patients (administrative data).

**Results:**

For medical admissions, higher mortality was associated with more occupied beds per RN (RR 1.22, 95% CI 1.04 to 1.43, p=0.02) and per doctor (RR 1.10, 95% CI 1.05 to 1.15, p <0.01) employed by the trust whereas, lower HCSW staffing was associated with lower mortality (RR 0.95, 95% CI 0.91 to 1.00, p=0.04). In multivariable models the relationship was statistically significant for doctors (RR 1.08, 95% CI 1.02 to 1.15, p=0.02) and HCSWs (RR 0.93, 95% CI 0.89 to 0.98, p<01) but not RNs (RR 1.14, 95% CI 0.95 to 1.38, p=0.17). Trusts with an average of ≤6 patients per RN in medical wards had a 20% lower mortality rate compared to trusts with >10 patients per nurse (RR 0.80, 95% CI 0.76 to 0.85, p<0.01). The relationship remained significant in the multivariable model (RR 0.89, 95% CI 0.83 to 0.95, p<0.01). Results for surgical wards/admissions followed a similar pattern but with fewer significant results.

**Conclusions:**

Ward-based RN staffing is significantly associated with reduced mortality for medical patients. There is little evidence for beneficial associations with HCSW staffing. Higher doctor staffing levels is associated with reduced mortality. The estimated association between RN staffing and mortality changes when medical and HCSW staffing is considered and depending on whether ward or trust wide staffing levels are considered.

**Strengths and limitations of this study**

- Most previous work has been concentrated in North America with few papers based on UK data.
- Like much of the research in this field, it uses a cross-sectional observational design and reports association (so cannot demonstrate causation).
- This study makes a unique contribution by including medical and healthcare support worker staffing in examining the observed relationships between trust staffing and mortality.
- The inclusion of medical staffing data however creates a limitation, in that the quality of the data available in England is restricted to posts: bed ratios.

## Introduction

Ensuring the safety of hospital care is a paramount concern for healthcare systems worldwide. Despite increasing expenditure and focus on patient safety in many countries, there remains considerable variation in hospital trust mortality that cannot be explained by measurable variation in case mix or individual patient risk.^1^ ^2^ Registered nurse (RN) staffing has been identified as an important modifiable factor that is associated with mortality in many studies across the world.^3–5^ A higher level of registered nurse staffing is associated with lower mortality and better quality of care. The strength of association varies across studies and settings, but a 6% increase in the odds of death associated with one additional patient per nurse is typical.^5^ ^6^ Findings such as these have informed policies mandating minimum nurse patient ratios in some US and Australian states.^7^ However, despite the apparently strong evidence base, the implications of the findings remain contested by many and there remains significant resistance to mandated ratios from politicians and healthcare providers in many countries.^8^ ^9^ Economic pressures and the ageing profile of the nursing workforce internationally all point to a potential future with fewer registered nurses.^10^ Current plans for workforce development in England and other countries point toward a significant increase in the numbers and proportion of unregistered support workers and assistant practitioners, relative to the number of registered nurses and registered nurse recruitment remains problematic.^11^ ^12^

However, such a shift seems to be at odds with evidence that points toward a more highly trained nursing workforce being associated with fewer adverse events.^13^ Research from the US and Europe showed that having a higher proportion of degree qualified nurses in the workforce was associated with lower surgical mortality rates,^5^ ^14^ ^15^ while cross-sectional studies in England have found that hospitals with more unqualified nurses per bed^16^ and a higher proportion of support staff to registered nurses^17^ had higher mortality rates. Both these English studies also showed a significant negative association between staffing by medical doctors and mortality rates; higher medical staffing levels were associated with lower mortality rates.^16^ ^17^ Indeed, the associations between registered nurse staffing and mortality were not significant when medical staffing was included in multivariable analyses. These studies have limitations. Both used organisation level staffing data, which may not reflect the deployment of staff on wards. The Keogh review, undertaken to explore higher than expected mortality rates in 14 NHS trusts, revealed a discrepancy between the view of nurse staffing levels gained from administrative data (full-time equivalent, FTE per bed) versus observing nurse staffing ‘on the ground’.^18^

None the less, these studies serve to illustrate that a failure to consider other staff groups concurrently is a significant limitation in much of the existing research on this topic. The boundaries between the work of different staff groups is fluid and there is some potential for the work of one group to substitute to some degree for that of another. For example, there is some evidence that substitution between nurses and doctors may be cost-effective in a variety of settings^19^ and in the UK for example, responsibilities have passed from doctors to nurses as the working hours of hospital doctors have reduced in response to EU legislative changes.^20^ On the other hand, unqualified support workers can undertake both clerical work and some aspects of clinical nursing care.^20^

This study therefore aims to determine the association between mortality and trust level registered nurse staffing in English general acute NHS hospital trusts while simultaneously considering staffing by support workers and doctors using routinely collected administrative data. Since routine data on ward level staffing is not widely available in national data sources, we also use ward level nurse data from a nationally representative subsample of trusts, derived from the RN4CAST survey of nurses^21^ to estimate nurse staffing actually deployed on wards.

## Methods

### Data sources

We obtained details of the workforce characteristics of NHS acute hospital trusts providing inpatient general medical and surgical care from the annual NHS staff census for 2009/2010 and 2010/2011. We excluded specialist trusts (eg, cancer, paediatrics), mental health trusts and trusts with low numbers of general medical/surgical admissions. We obtained details of teaching status, bed occupancy and number of beds from the annual estates and facilities statistics for 2009/2010 and 2010/2011. From this, we calculated ratios of beds per RN, doctors and healthcare support workers (HCSWs including healthcare assistants and auxiliary nurses). HCSWs in England are unregistered care staff (without nursing qualifications) who undertake many aspects of fundamental care for patients in NHS hospital wards (such as helping patients to wash, use the toilet, and monitoring vital signs). Patient data were obtained from the national Hospital Episode Statistics for patients admitted in the 2 years from 1 April 2009 to 31 March 2011. We were able to link trust level staffing, bed occupancy and mortality data for 137 trusts. The census data does not specifically identify nurses employed delivering inpatient care on wards. Therefore, in addition to the data derived from routinely collected data sets, we also assessed nurse staffing on medical and surgical wards directly for a nationally representative subsample of 31 trusts, by means of a survey of nurses from a stratified random sample of general medical/surgical wards (up to 10) in each hospital in the trust. The survey was undertaken from January to September 2010 as part of the RN4CAST study. RNs in the 31 trusts (covering 46 hospitals and 401 wards) were surveyed; 2990 of 7609 (39%) responded. The nurse response rate varied between the 31 trusts from 19% to 69%.

Nurses reported on patient and staff numbers present on their last shift. Patients per RN and patients per HCSW were calculated for each nurse responding to the survey. Staffing levels (patients per nurse) for the medical and surgical wards of each hospital trust were estimated by averaging responses from all nurses in each specialty. Wards classified as mixed medical/surgical were treated as medical. Detail of the design and methods of this survey reported elsewhere.^21^ ^22^

### Risk-adjusted mortality

We calculated the predicted number of deaths in hospital trusts for both medical and surgical admissions, using a method based on that used to calculate the summary mortality Indicator in England.^23^ This uses indirect standardisation for age, sex, elective status, socioeconomic deprivation (Index of multiple deprivation), comorbidity (modified Charlson Index) and number of emergency admissions in the previous 12 months. We collapsed reasons for admission into the Clinical Classifications Software (CCS) groupings given by the Agency for Healthcare Research and Quality.^24^ For each CCS group we built a logistic regression model to predict the probability of death. We divided admissions into medical and surgical specialties using the specialty code of the admitting consultant and calculated the predicted number of deaths in each group for each trust by summing the predicted number of deaths across all CCS groups. Thus we were able to assess the risk of deaths in a trust relative to the number that would be expected given the case mix.

### Analysis data set

Data consisted of observed and expected deaths aggregated by medical and surgical specialty for 2009–2010 and 2010–2011 separately. These data were linked to trust level staffing data, hospital trust size and teaching status for each year.

### Analysis

We used the Generalised Estimating Equations (GEE) modelling procedure in SPSS V.22 to produce crude and adjusted effects of staffing on mortality. GEE was used in preference to a multilevel model because it is more suited to estimating population average effects. There were only two time-points, which would have limited the usefulness of a multilevel model. Observed deaths were regressed on the independent variables and the natural log of the expected number of deaths was used as an offset. All adjusted staffing effects controlled for hospital trust size (bed numbers), admission year and teaching status.

For the national (137 trusts) analysis we calculated ratios of staff per occupied bed at the hospital trust level and used mortality and staffing data for 2009–2010 and 2010–2011. For the analysis of the subsample (n=31) we used data from 2010–2011 only (to most closely match when the survey was in the field) and used estimates of RN per patient and HCSW per patient for medical and surgical units derived from ward staffing reported in our survey to model associations with medical and surgical mortality, respectively. Ward-based RN staffing levels were modelled in four groups (in medical ≤6 (n=2), 6.01–8.00 (n=13), 8.01–10.00 (n=13) and ≥10 (n=2); in surgical ≤6 (n=6), 6.01–8.00 (n=16), 8.01–10.00 (n=8) and ≥10 (n=1)). Since no equivalent ward-based measure of medical staffing was available we retained hospital trust level doctors per bed to control for medical staffing in this analysis.

An assessment of collinearity was performed prior to fitting the GEE models. If the condition index was 30 or greater the independent variables would be further scrutinised using the variance inflation factor and variance proportions.^25^ ^26^ Consideration was then given to removing variables causing the collinearity from the model. The condition index was below 30 for all models without interactions. However, when interactions (eg, occupied beds per FTE RN x occupied beds per FTE HCSW) were added the condition indices exceeded 100 and so interactions were excluded from the models.

## Results

In the 137 hospital trusts there were 9 669 555 medical admissions and 9 302 292 surgical admissions over 2 years, with overall death rates of 32.8 and 7.9/1000, respectively. There was substantial variation between trusts in medical and nurse staffing with a more than fourfold variation in registered nurse staffing between the lowest and highest staffed hospital trust. This was attenuated when considering all nursing staff (RN + HCSW), although the variation was still more than threefold. These large variations are reflected in the 31 trusts where we had measures of nurse staffing on wards, where variation between highest and lowest staffed ranged from 2 to 2.5 times across staff groups and specialties (table 1).

**Table 1:** Staffing levels (full-time equivalents)

	Mean	Minimum	Maximum
*137 trusts 2009–2011 (hospital trust level employed staff, full-time equivalents)*			
Occupied bed per RN	1.53	0.69	2.81
Occupied bed per HCSW	0.67	0.31	1.14
Occupied bed per nurse (HCSW+RN)	2.20	1.09	3.45
Occupied bed per Doctor	0.74	0.35	1.30
*31 trusts 2010 (ward staff)*			
Medical wards			
Patient per RN	7.97	4.85	11.06
Patient per HCSW	8.92	5.48	13.14
Patient per nurse (HCSW+RN)	4.15	2.68	5.61
Surgical wards			
Patient per RN	7.33	4.60	11.34
Patient per HCSW	9.58	5.72	14.68
Patient per nurse (HCSW+RN)	4.10	2.59	5.21

HCSW, healthcare support worker; RN, registered nurse.

The correlations between staffing variables were typically weak to moderate although there was a strong correlation between occupied beds per FTE RN and occupied beds per FTE Doctor (r=0.72; table 2).

**Table 2:** Correlations between staffing variables

137 Trusts	Occupied beds per FTE HCSW	Occupied beds per FTE Doctor
Occupied beds per FTE RN	0.13, p=0.031	0.72, p<0.001
Occupied beds per FTE HCSW		−0.14, p=0.021

31 RN4CAST trusts	Patients per HCSW	Occupied beds per FTE Doctor

Patients per RN	0.38, p=0.002	−0.40, p=0.001
Patients per HCSW		−0.24, p=0.056

HCSW, healthcare support worker; FTE, full-time equivalent; RN,registered nurse.

### Whole trust staffing

In the unadjusted analysis for medical admissions, an increase in the number of occupied beds per whole time equivalent RN (RR 1.22, p=0.016) and doctor (RR 1.10, p<0.001) were associated with an increase in mortality. For HCSW this association was reversed (RR 0.95, p=0.041). In the adjusted analysis the association for RNs was attenuated and no longer statistically significant (RR 1.14, p=0.17), but remained statistically significant for doctors (RR 1.08, p=0.016) and for HCSWs (RR 0.93, p=0.003; table 2).

For surgical admissions, neither occupied beds per RN (RR 1.15, p=0.088) nor HCSW (RR 0.96, p=0.20) were significantly associated with mortality although the direction of the associations were similar to that for medical admissions. An increase in the number of occupied beds per FTE Doctor was significantly associated with an increase in mortality (RR 1.08, p=0.020). In the adjusted model the association with occupied beds per FTE Doctor strengthened (RR 1.13, p=0.002), but remained non-significant for RNs (RR 0.94, p=0.59) and HCSWs (RR 0.95, p=0.22; table 3).

**Table 3:** Association between trust level staffing and standardised mortality: 137 NHS trusts

	Unadjusted				Adjusted			
Parameter	Risk ratio	L_95% CL_	U_95% CL_	p Value	Risk ratio	L_95% CL_	U_95% CL_	p Value
Medical								
Non-teaching trust					1.03	0.96	1.09	0.43
Year, 2009/2010					0.99	0.98	1.01	0.26
Beds (thousands)					0.98	0.93	1.03	0.43
Occupied beds per FTE RN	1.22	1.04	1.43	0.016	1.14	0.95	1.38	0.17
Occupied beds per FTE HCSW	0.95	0.91	1.00	0.041	0.93	0.89	0.98	0.003
Occupied beds per FTE Doctor	1.10	1.05	1.15	<0.001	1.08	1.02	1.15	0.016
Surgical								
Non-teaching trust					1.01	0.94	1.09	0.71
Year, 2009/2010					0.97	0.95	1.00	0.02
Beds (thousands)					1.05	0.97	1.14	0.25
Occupied bed per FTE RN	1.15	0.98	1.36	0.088	0.94	0.73	1.20	0.59
Occupied beds per FTE HCSW	0.96	0.89	1.02	0.20	0.95	0.88	1.03	0.22
Occupied beds per FTE Doctor	1.08	1.01	1.16	0.020	1.13	1.04	1.22	0.002

HCSW, healthcare support worker; FTE, full-time equivalent; NHS, National Health Service; RN, registered nurse.

### Ward-based nurse staffing

In our subsample of 31 trusts where we used a survey to measure nurse staffing on medical and surgical wards, mortality rates were similar to the national sample with 35.2 deaths/1000 medical admissions (total medical admissions 1 260 558) and 8.9 deaths/1000 surgical admissions (total surgical admissions 1 084 429). All staffing variables were significantly associated with mortality in the unadjusted analysis (p<0.01, table 4).

**Table 4:** Association between ward level staffing and standardised mortality: 31 trusts

	Unadjusted				Adjusted			
	Risk ratio	L_95% CL_	U_95% CL_	p Value	Risk ratio	L_95% CL_	U_95% CL_	p Value
Medical								
Non-teaching trust					1.12	1.08	1.15	<0.01
Beds (thousands)					1.08	1.04	1.13	<0.01
Patients per RN (χ^2^, df, p value)	(59.831, 3df, <0.001)				(12.524, 3df, <0.001)			
≤6	0.80	0.76	0.85	<0.001	0.89	0.83	0.95	0.001
6.01–8.00	0.92	0.87	0.96	<0.001	0.96	0.91	1.01	0.14
8.01–10.00	0.91	0.87	0.96	<0.001	0.96	0.91	1.01	0.11
≥10	1.00				1.00			
Patients per HCSW	1.01	1.00	1.02	0.001	1.00	0.99	1.01	0.92
Occupied beds per FTE Doctor	1.24	1.19	1.28	<0.001	1.12	1.06	1.17	<0.001
Surgical								
Non-teaching trust					1.09	1.03	1.17	<0.01
Beds (thousands)					1.15	1.07	1.24	<0.01
Patients per RN (χ^2^, df, p value)	(11.604, 3df, 0.009)				(3.290, 3df, 0.349)			
≤6	0.83	0.69	1.00	0.049	0.89	0.73	1.08	0.23
6.01–8.00	0.90	0.75	1.08	0.26	0.90	0.75	1.09	0.30
8.01–10.00	0.90	0.75	1.08	0.26	0.87	0.73	1.05	0.16
≥10	1.00				1.00			
Patients per HCSW	1.02	1.01	1.03	0.002	1.01	1.00	1.03	0.053
Occupied beds per FTE Doctor	1.22	1.13	1.31	<0.001	1.15	1.03	1.28	0.010

HCSW, healthcare support worker; FTE, full-time equivalent; RN, registered nurse.

Mortality was higher in trusts where RNs cared for more patients. Trusts with six or less patients per RN in medical wards had a 20% lower risk of death among medical patients compared to trusts with over 10 patients per nurse (RR 0.80, p<0.001). The corresponding reduction for surgical wards/patients was 17% (RR 0.83, p=0.049). This difference was attenuated but remained significant in the adjusted model for medical wards (RR 0.89, p<0.001) but not for surgical wards (RR 0.89, p=0.23) (table 4).

Every additional patient per HCSW was associated with a 1% increase in mortality for medical patients (RR 1.01, p=0.001) and a 2% increase for surgical patients (RR 1.02, p=0.002). These adjusted associations were attenuated and non-significant, although on surgical wards this association neared statistical significance (RR 1.01, p=0.053; table 4).

The unadjusted associations with occupied beds per FTE doctor were stronger in this subsample than in the 137 trusts. These associations were significant in the unadjusted (medical RR 1.24, p<0.001; surgical RR 1.22, p<0.001) and adjusted analyses (medical RR 1.12, p<0.001; surgical RR 1.15, p=0.010; table 4).

## Discussion

In this study, we assessed associations between RN staffing and mortality using both national administrative staffing data and surveys of ward level staffing in a subsample. We simultaneously considered staffing by medical doctors and support workers (HCSW). When all staff groups were included (in the analysis of 137 hospital trusts) the adjusted associations with mortality were not statistically significant for nurse staffing but were for doctor staffing. In our subsample higher nurse staffing levels were significantly associated with lower mortality among both medical and surgical patients in the adjusted model. Higher HCSW staffing was associated with higher levels of risk adjusted mortality in the analysis of 137 trusts. In the subsample, which used nurse survey-based estimates of HCSW staffing levels, the adjusted association was not significant.

Although the evidence showing associations between higher RN staffing and reduced mortality is extensive, few previous studies have considered staffing by both doctors and HCSW while exploring the relationship and none has done so using estimates of ward-based nurse staffing. Previous studies using hospital trust level data found little evidence for a relationship between RN staffing and mortality adjusting for medical staffing^16^ ^17^ although one US study, which did not include HCSW staffing, found a significant relationship for RN staffing after adjusting for medical staffing.^27^ A study of ICUs in England found a relationship between consultant numbers, RN numbers and mortality, but no evidence of a relationship with support worker levels.^28^ Other studies which have considered less highly qualified nursing staff in hospitals (licensed practical nurses and unlicensed support workers) have shown higher numbers of less trained staff or a diluted nursing skill mix to be associated with higher mortality or lower cost-effectiveness.^16^ ^17^ ^29^ In our study the negative relationship was not replicated when considering estimates of ward-based HCSW staffing. However, a challenge in interpreting study findings, is the extent to which the role of the Health Care Support Worker or ‘nursing aide’ role varies.^30^

This illustrates that the source of data used to explore these associations is an important consideration. Inferences about ward staffing made from hospital or trust level data may be incorrect.

There is currently significant debate about establishing mandatory minimum nurse staffing levels in England and elsewhere. However, the evidence base to draw on in order to identify specific safe staffing ratios is slim, despite the large volume of research. Recommended or mandated staffing levels for RNs in general medical and surgical units range from no more than four patients per RN (day shift in level 1 hospitals in the State of Victoria, Australia) to 10 patients per RN at night (level 2/3 hospitals in Victoria). Ratios between 4–1 and 6–1 on day shifts are typical.^31^ In this study, irrespective of specialty, the risk of mortality was 11% lower in trusts where registered nurses reported caring for an average of 6 or fewer patients compared to trusts where nurses reported caring for an average of 10 or more.

Although the pattern of results for medical and surgical mortality were similar, we did not find significant adjusted associations between registered nurse staffing and surgical mortality, using either the trust-wide or nurse estimated ward staffing. In previous research the relationship between RN staffing and surgical patient outcomes has been clearer than for medical patients.^32^ We used all surgical admissions in our study, where overall mortality rates are low, whereas previous research has typically focused on high-risk subgroups of patients, which may provide a more sensitive indicator.

Although policy in England has raised the possibility of using HCSW to substitute for RNs, the evidence here suggests that this may not be consistent with patient safety. We found that trusts with more HCSWs per bed had higher rates of mortality among medical patients. Although this finding was not replicated when we looked at nurse estimated ward staffing levels, our adjusted models showed no evidence for benefit from higher HCSW staffing levels. This is consistent with other findings from the RN4CAST study which found no association between the level of HCSW staffing and the occurrence of missed nursing care reported by RNs.^22^ While HCSW may deliver essential care, there is no evidence from large observational studies that their presence in the workforce can substitute for registered nurses in ensuring patient safety.

In common with most research in this area our study was cross-sectional and cannot demonstrate causation, although the association between nurse staffing and mortality has recently been demonstrated in a prospective study.^33^ Our study has several limitations; the ward-based staffing data arises from only 31 trusts and was estimated from nurse report. This does not, in itself, provide a robust basis to identify safe staffing thresholds. Although we had ward level staffing data, it was only possible to model outcomes at the level of medical/surgical specialties rather than at the level of the ward, and therefore any variation at the ward level remains hidden. Further research is required to provide more robust estimates of associations in larger samples of hospital trusts. Our results do not provide support for using HCSW to substitute for registered nurses but we were unable to consider whether they may act as complements, enhancing the effectiveness of RNs, because we were unable to explore the interaction between different staff groups due to collinearity. However, our previous work on nursing care left undone suggests that HCSWs neither substitute for nor complement the ability of RNs to deliver core professional nursing work.^22^

## Conclusions

Based on these findings we conclude that while a causal association between RN staffing and patient outcomes remains plausible, the current evidence base is not sufficient to identify safe staffing thresholds across different types of wards. However, given the overall strength of evidence for an association, it does seem feasible to identify staffing levels where risk to patients is likely to be increased, as recently suggested in a review of safety in the NHS.^34^ When determining the safety of nurse staffing on hospital wards, the level of RN staffing is crucial and there is no evidence to suggest that higher levels of HCSW staffing have a role in reducing mortality rates. Current policies geared toward substituting HCSW for registered nurses should be reviewed in the light of this evidence. Future research exploring associations between nurse staffing and patient outcomes needs to include measures of both medically qualified staff and unregistered practitioners.
